# Changes in Eating Attitudes, Body Esteem and Weight Control Behaviours during Adolescence in a South African Cohort

**DOI:** 10.1371/journal.pone.0109709

**Published:** 2014-10-13

**Authors:** Tabither M. Gitau, Lisa K. Micklesfield, John M. Pettifor, Shane A. Norris

**Affiliations:** Wits/MRC Developmental Pathways for Health Research Unit, Faculty of Health Sciences, University of the Witwatersrand, Johannesburg, South Africa; McMaster University, Canada

## Abstract

Failure to consume an adequate diet or over consumption during adolescence can disrupt normal growth and development, resulting in undesirable weight change. This leads to an increase in unhealthy weight control practices related to eating and exercise among both adolescent girls and boys to meet the societal ‘ideal’ body shape. This study therefore aims to examine the longitudinal changes in eating attitudes, body-esteem and weight control behaviours among adolescents between 13 and 17 years; and, to describe perceptions around body shape at age 17 years. A total of 1435 urban South African black and mixed ancestry boys and girls, who had data at both age 13 and 17 years from the Birth to Twenty cohort were included. Data were collected through self-administered questionnaires on eating attitudes (EAT-26), body esteem and weight control behaviours for either weight loss or muscle gain attempts. Height and weight were measured at both time points and BMI was calculated. Black females had a higher BMI (p<0.001) and an increased risk of developing eating disorders as well as significant increase in the prevalence of weight loss practices between the ages 13 and 17 years. At age 17 years both Mixed ancestry adolescents had lower body-esteem compared to black adolescents. The prevalence of possible eating disorders was 11% and 13.1% in early and late adolescents respectively. Males and females shared similar opinions on normal silhouettes being the ‘best’, ‘getting respect’ and being the ‘happiest’, while the obese silhouette was associated with the ‘worst’ and the ‘unhappiest’, and the underweight silhouette with the “weakest”. Black females had a higher BMI and an increased risk of developing eating disorders. Adolescent females engaged more in weight loss practices whereas, males in muscle gain practices indicating that Western norms of thinness as the ideal are becoming more common in South Africa.

## Introduction

Nutritional needs are greater during adolescence than at other times during childhood, and over-consumption or failure to consume an adequate diet during this period can disrupt normal growth and development, resulting in undesirable weight change [1] Previous research has shown that unhealthy weight control practices related to eating and exercise are on the rise among adolescent girls and boys in order to meet the societal ‘ideal’ body shape, particularly in the developed world [2], [3].

In most high-income countries (HICs) [4] women are considered desirable, attractive, and successful when they are lean [5]. Furthermore, men are considered attractive when they have a muscular body shape with large shoulders and a slim waist [6]. It is during adolescence that individuals start to experience body dissatisfaction, and may begin engaging in unhealthy weight control behaviors such as fasting, skipping meals, excessive dietary restriction, consuming diet pills, self-induced vomiting and extreme exercising [2]. In contrast, in some African cultures being overweight is associated with positive attributes [7]. In South African women, leanness is not necessarily perceived as beautiful; rather being plump (overweight) signifies beauty, health and a higher social status [8]. Furthermore, black African women associate overweight with happiness, affluence, and the absence of disease (such as HIV/AIDS), and having overweight children is a measure of ‘good’ parental care [8]. However, with ongoing Westernization occurring in South Africa [9], there has been a change in eating attitudes and body image satisfaction in South African adolescents.

Research by Mchiza and others in an urban adolescent sample (Cape Town) of South African girls found that body image dissatisfaction was greater in white than black African girls. In addition, black African girls experienced less pressure from family and peers to change their current body shape [10]. In another South African study of 15–18 year-olds, 33%, 26% and 20% of white, black African and mixed ancestry girls, respectively experienced body dissatisfaction [11]. More recently in a cross sectional study in urban South Africa (Soweto) we found that more black African than white girls were at risk of future eating disorders, however the black adolescents still favored a body silhouette with a greater BMI than their white peers [12].

Some studies have shown that increasing age during adolescence is strongly associated with heightened emphasis on appearance and body shape [13], however other studies have shown a strong association between low self-esteem and body dissatisfaction across gender, age, weight status, ethnicity, socioeconomic status and time. [14].

Because of limited longitudinal information on eating attitudes, body image perception and weight control behaviors in South African black and mixed ancestry adolescents, we examined changes in these parameters between early (13 years) and late (17 years) adolescent boys and girls. Secondly, we assessed differences between 17-year-old boys and girls in their perceptions of female body silhouettes.

## Methods

### Sample and study design

Data for this study were obtained from the Birth-to-Twenty longitudinal birth cohort study [15]. A total of 3 723 babies born to mothers who were residing in the Soweto-Johannesburg (formal and informal urban areas) region for at least 6 months after birth in 1990 were enrolled into the study. After nearly two decades, the cohort has had a relatively low attrition rate of 30%, and approximately 2 300 participants still remained in contact with the study [15].

A trained research team collected socio-demographic and anthropometric data, and interviewer-assisted questionnaires on eating attitudes, body esteem and weight control behaviors on the adolescents at two time points: at age 13 years (n = 1 580: girls = 833, boys = 747) and four years later at age 17 years (n = 1 820: girls = 939, boys = 881). A total of 1 435 participants had complete data at both 13 and 17 years (boys = 690 and girls = 745). This study only included black African and mixed ancestral (children born to white European and black African parents) participants from the cohort and those who had data at both time points. Adolescents belonging to other racial groups (i.e. white and Indian) were excluded because of the small sample sizes. This study is presented in two-phases: A cross- sectional study at age 13 and 17 years, and a longitudinal study that examines the changes that took place between age 13 and 17 years. Ethics approval was obtained from the University of the Witwatersrand Human Research Ethics Committee. Written informed consent was obtained from the participants’ legal guardians/caregivers, and written assent from the participants themselves.

### Measures

Height and weight were collected at both time points. Height was measured using a portable stadiometer (Holtain; UK) and recorded to the nearest millimeter, and weight was measured using a digital scale to the nearest 100 grams. Participants’ height and weight measurements were used to calculate BMI (weight (kg)/height^2^ (m^2^)), and an international age and gender BMI cut-off were used to define overweight, obesity [16] and thinness prevalence (2^nd^ grade i.e. <2SD) [4].

The EAT-26 questionnaire was completed in order to measure eating attitudes [17]. This questionnaire has been previously validated in both rural [18] and urban [18] South African settings. The total EAT-26 score is the sum of the 26 items and scores range from 0 to 78. Participants who score more than 20 are considered to be at greater risk of developing an eating disorder, and represent more unhealthy attitudes towards food, body weight and eating. We utilized Cronbach alpha to determine the internal reliability for EAT-26 at both early and late adolescence and found that the questionnaire had good reliability (early adolescence: α = 0.71 and late adolescence: α = 0.70).

Body-esteem was measured using a body esteem scale [19]. It consists of a set of 21 questions which measure 1) global feelings about one’s body e.g. “I like what I see when I look in the mirror”, 2) satisfaction with one’s weight e.g. “I really like what I weigh” and 3) positive evaluations about one’s body and appearance e.g. “People my own age like my looks”. The body-esteem assessment uses a 5-point scale ranging from “never” (1) to “always” (5) and the higher the score the more satisfied the participant is with their body. Total scores are divided into three categories; low body esteem (score 1 to 21), average body-esteem (score 22 to 42), and high body esteem (score≥43) [19]. Internal reliability for the body-esteem scale was very good (early adolescence: α = 0.86 and late adolescence: α = 0.89).

All participants were asked a number of questions about their attempts to change their weight. Girls and boys were asked the following questions: “During the past year have you done anything to try to lose weight?”, “During the past year have you done anything to try to gain muscle?” If participants answered positively they were asked to give reasons, which included; health and cosmetic reasons e.g. to look better, clothes too tight, too fat, unhappy with self, and want to be a model. They were further asked about the methods they used to lose weight, and their responses were categorized into three groups: (i) healthy weight control behaviors (e.g. to exercise, eat more fruits and vegetables, and to eat less high fat foods and less sweets) [20] unhealthy weight control behaviors (e.g. fasting, eating very little food, skipping meals, cigarette smoking and use of food substitutes (iii) extreme weight control behaviors (e.g. use of diet pills, self-induced vomiting, use of laxatives and diuretics).

At 17 years of age girl and boy participants were also asked to select a female body silhouette from a series of 8 randomly arranged body silhouettes [19] which they associated with the following specific words or phrases: ‘looks best’, ‘looks clumsy’, ‘looks worst’, ‘looks happy’, ‘looks strong’, ‘looks weak’, ‘I respect’, ‘looks unhappy’. For the purposes of this analysis the body silhouettes were coded from 1 (thinnest) to 8 (biggest) and then grouped into 4 categories; silhouettes 1 and 2 (underweight), 3 and 4 (normal), 5 and 6 (overweight) and 7 and 8 (obese).

### Data analysis

STATA (Version 12 StataCorp, Texas USA) was used for analysis. Skewness and kurtosis tests for normality were applied to all numerical variables. A kurtosis of 3 was used to define a normal distribution and parametric tests were conducted. Data with a kurtosis greater than 3 were considered to be skewed and were subjected to non-parametric methods. None of the data was transformed. Student t-tests were used to determine differences in normally distributed numerical variables between the binary variables (e.g. gender and ethnicity). Associations between categorized weight–control behaviors and other characteristics (gender, ethnicity and BMI category) were assessed with chi-squared tests.

Multi-nominal logistic regression models were fitted to identify determinants of weight control behaviors, eating attitudes and body esteem. We ran several bivariate models and identified variables that were significantly associated with the outcomes at the 5% level and included them in the multinomial regression.

## Results

This study results are presented in three parts: Part 1 examines the cross-sectional data at ages 13 and 17 years, part 2 examines longitudinal changes in those who had complete data at both time points, and part 3 presents the results of the participants’ perceptions of the female silhouettes at age 17 years.

The prevalence of underweight across the four groups at age 13 years (table 1) was 2.1%, and 7.4%, in black African boys and girls, respectively and 3.6% and 6.6% in mixed ancestry boys and girls, respectively. Black African girls had the highest prevalence of overweight (14%) followed by mixed ancestry females (7.8%), black African boy adolescents (6.3%), and mixed ancestry boys (4.9%). Black African girls were significantly heavier (p = 0.012) and had a higher BMI (p = 0.014) than mixed ancestry girls, but there were no differences between the boys in weight or BMI (p>0.05), although the mixed ancestry boys were taller than the black African boys (p = 0.025).

**Table 1:** General characteristics of 13 year-old black African and mixed ancestral urban South_ African boys and girls._

	Black African			Mixed ancestral			P-value	
Variables	Boys (n = 666)	Girls (n = 742)	P-value	Boys (n = 81)	Girls (n = 91)	P-value	A	B
**Age (years)**	13.7±0.2	13.7±0.2	0.233	13.7±0.2	13.7±0.2	0.233	0.241	0.122
**Height (cm)**	154.5±8.4	155.7±6.2	**0.002**	156.8±9.9	155.1±6.7	0.188	**0.025**	0.346
**Weight (kg)**	44.6±10.1	50.2±11.5	**0.001**	44.7±11.3	47.0±10.9	0.16	0.977	**0.012**
**BMI (kg/m^2^)**	18.6±3.2	20.6±4.2	**0.001**	18.0±3.3	19.5±4	**0.008**	0.09	**0.014**
**BMI (kg/m^2^)** a			**0.001**			**0.008**	**0.04**	0.216
**Underweight**	14(2.1%)	27(3.6%)		6(7.4%)	6(6.6%)			
Normal	580(87.1%)	565(72.2%)		69(83.9%)	73(80.2%)			
Overweight	42(6.3%)	104(14%)		4(4.9%)	7(7.7%)			
Obese	21(3.2%)	46(6.2%)		2(2.5%)	5(5.5%)			
**EAT-26**	8(4–14)	8(3–14)	0.625	9(4–13)	7(4–12)	0.779	0.599	0.831
**EAT-26 score**						0.608	0.676	0.605
<20	594(89.2%)	655(88.3%)	0.588	71(87.7%)	82(90.1%)			
>20	72(10.8%)	87(11.7%)		10(12.3%)	9(8.9%)			
**Body-esteem**								
Low	1(0.1%)	0%	0.332	0%	0%	0.54	0.377	0.551
Average	636(95.5%)	717(96.6%)		80(98.8%)	89(97.8%)			
High	29(4.4%)	25(3.4%)		1(1.2%)	2(2.2%)			
**Weight loss practices**								
No	575(86.5%)	599(80.7%)	0.004	74(91.4%)	69(75.8%)	0.008	0.216	0.268
Yes	90(13.5%)	143(19.3%)		7(8.6%)	22(24.2%)			
**Muscle gain practices**			0.023			0.028	0.906	0.268
No	335(50.6%)	644(88.1%)		36(45%)	69(76.7%)			
Yes	327(49.4%)	87(11.9%)		44(55%)	21(23.3%)			
**Weight control behaviours**						0.292	0.573	0.69
Healthy	23(56.1%)	58(58.6%)		4(80%)	8(53.3%)			
Unhealthy	16(39.0%)	38(38.4%)		1(20%)	7(46.7%)			
Extreme	2(4.9%)	3(3.0%)						

a Cole et al Age-gender specific BMI cutoffs for age 13.5 years adolescent boys and girls.

A- Statistical significance for black African and Mixed ancestral boys.

B – Statistical significance for black African and Mixed ancestral girls.

In the whole group, 11% of the participants reported an EAT-26 score >20, which is indicative of being at risk of developing an eating disorder, however there were no sex or ethnic differences between the groups. There was also no difference between the genders or ethnic groups for those adolescents categorized as low, average or high body esteem. Significantly more girls than boys engaged in weight loss practices (p<0.05), and conversely, significantly more boys than girls engaged in muscle gain practices (p<0.05).

A significant proportion of 13 year olds engaged in unhealthy weight control behaviors (boys 37% and girls 39.5%), with the majority (58%) engaging in healthy weight control habits.

The prevalence of underweight among the 17 year-old adolescents ranged from 2.7–8.6% in the girls and 6.2–10% in the boys (table 2). Overweight prevalence ranged from 9.7–19.1% in the girls, and 4–4.5% in the boys, and obesity prevalence ranged from 6.2–8.4% in the girls and 1.8–7% in the boys. Black African girls weighed more (p = 0.001) and had a higher BMI (p = 0.0001) than the mixed ancestry girls, and there was a significant difference in the proportion of underweight, normal weight, overweight and obesity between the ethnic groups for the boys (p = 0.005) and the girls (p = 0.001).

**Table 2:** General characteristics of 17 year old black African and mixed ancestral urban South_ African boys and girls._

Variables	BlackAfrican			Mixed ancestral				P-value	
Age (years)	Boys(n = 781)	Girls(n = 826)	P-value	Boys(n = 100)		Girls(n = 113)	P-value	A	B
**Height (cm)**	17.7±0.3	17.7±0.3	0.342	17.9±0.3		17.9±0.3	0.456	0.465	0.435
**Weight (kg)**	59.3±9.7	58.9±12	0.513	59.9±13.9		54.1±11.9	**0.001**	0.57	**0.001**
**BMI (kg/m^2^)**									
**BMI (kg/m^2^)** a	20.3±2.9	23.1±4.5	**0.001**	20.4±4.2		21.3±4.5	0.101	0.713	**0.001**
Underweight	48(6.2%)	23(2.7%)	**0.001**	10(10%)		10(8.6%)	0.131	0.005	**0.001**
Normal	684(87.6%)	575(66.1%)		79(79%)		85(72.7%)			
Overweight	35(4.5%)	158(19.1%)		4(4%)		11(9.7%)			
Obese	14(1.8%)	69(8.4%)		7(7%)		7(6.2%)			
**EAT-26**	9(6–15)	10(6–17)	0.193	9(3–14)		9(6–13)	0.91	0.779	0.493
**EAT-26 score**									
<20	661(84.6%)	689(83.4%)	0.504	90(90%)		101(89.5%)	0.882	0.154	0.104
>20	120(15.4%)	137(16.6%)		10(10%)		12(10.5%)			
**Body-esteem**							0.833	**0.001**	**0.001**
Low	26(3.3%)	29(3.4%)	0.85	11(11%)		14(12.3%)			
Average	785(95%)	785(95%)		89(89%)		100(87.7%)			
High	9(1.1%)	12(1.6%)		0(0%)		0%			
**Weight loss practices**			0.001				0.307	0.513	0.005
No	417(55.2%)	704(88.2%)		77(86.5%)		81(81%)			
Yes	339(44.8%)	94(11.8%)		12(13.5%)		19(19%)			
**Muscle gain practices**			0.001				0.001	0.006	0.703
No	417(55.2%)	704(88.2%)		52(58.4%)		84(84%)			
Yes	339(44.8%)	94(11.8%)		37(41.6%)		16(16%)			
**Weight control behaviors**			0.001				0.405	0.434	0.299
Healthy	52(81.2%)	118(63.4%)		5(71.4%)		5(41.7%)			
Unhealthy	8(12.5%)	55(29.6%)		2(28.6%)		6(50%)			
Extreme	4(6.3%)	13(6.7%)				1(8.3%)			

a Cole et al Age-gender specific BMI cutoffs for age 17.5 years adolescent boys and girls.

A- Statistical significance for black African and mixed ancestral boys.

B – Statistical significance for black African and mixed ancestral girl.

For the whole sample at age 17 years, 13.1% had an EAT-26 score >20, and there was no difference in EAT-26 score or the proportion of adolescents with an EAT-26 score >20 between the gender or ethnic groups. Significantly more mixed ancestry boys and girls had low body esteem compared to their black African counterparts (both p = 0.001), however there were no differences within the ethnic groups. Significantly more black African girls than boys engaged in weight loss practices (p = 0.001) and significantly more boys than girls, in both ethnic groups, engaged in muscle gain practices (both p = 0.001). In addition, there was a difference between the black and mixed ancestry boys with regard to participation in muscle gain practices. For the whole group, 14% of the boys and 30.8% of the girls engaged in unhealthy weight control behaviors, however there were no significant differences between the gender and ethnic groups for weight control behaviours.

**Figure 1:**
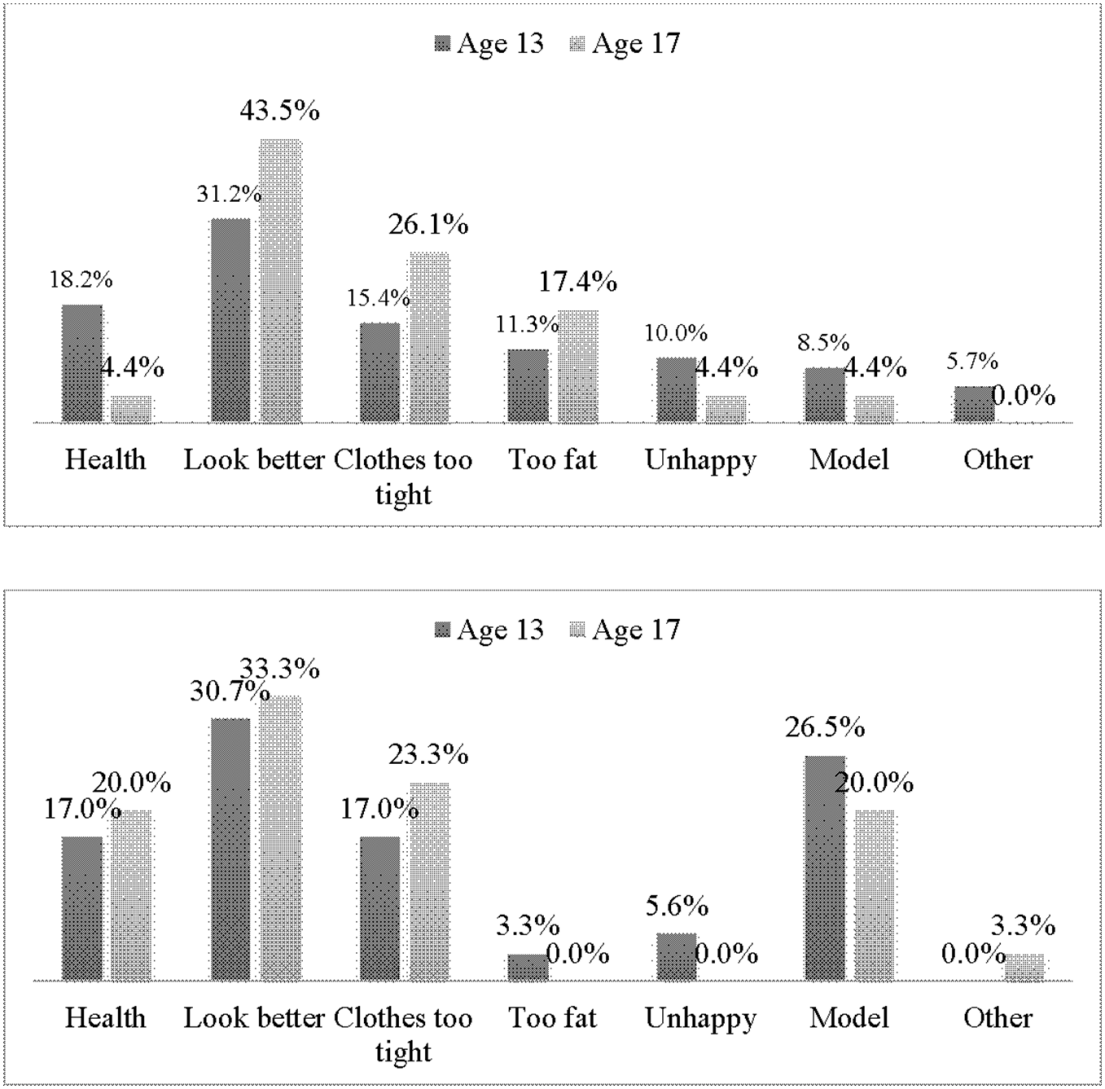
Reasons for weight control behaviors among age 13 and 17 black African girls (A); black African boys (B); mixed ancestral girls (C) and mixed ancestral boys (D).

The frequency of the various reasons given for the weight control behaviors reported by the adolescents at 13 and 17 years of age was determined (Figures 1A–D). The desire to look better was the most commonly reported reason given by all four groups at both time points, in both girl ethnic groups at age 13 and 17 years, another reason included clothes being too tight, and the mixed ancestry girls also wanted to control their weight for health reasons (11.1% of the 13 year olds and 25% of the 17 year olds). The main reasons given by the black African boys at both time points were for health reasons, a desire to look better, their clothes being too tight and desire to model. Among the mixed ancestry boys at 17 years of age, 50% of them engaged in weight control behaviors because they wanted to look better and to model, while the majority of the mixed ancestry 13 year old boys engaged in weight control practices mainly for health reasons (12.5%), a desire to look better (34.4%) and because their clothes were too tight (34.4%).

### Factors associated with eating attitudes, weight control behaviors and body esteem

At 13 years of age, there was no significant association between body esteem, eating attitudes and weight control behaviors, and ethnicity and BMI. At 17 years old, girls were more likely than boys to engage in unhealthy weight control behaviours (OR 1.05, 95% CI: 0.037–1.72, p = 0.002) irrespective of ethnicity and BMI. At age 17 years girls of mixed ancestry origin were 36% less likely to be at risk of developing eating disorders (EAT26>20) compared to their black African peers, irrespective of BMI (OR −0.64, 95% CI: −1.26-−0.036, p = 0.038).

### Longitudinal change in eating attitudes, body esteem and weight control behaviors between 13 and 17 years of age (N = 1,435)

Table 3 presents the longitudinal changes in BMI category (underweight, normal weight, overweight and obese), EAT-26 (>20), body-esteem (low, average or high), weight loss practices (yes or no) and muscle gain practices (yes or no) in black African and mixed ancestry boys and girls between the age of 13 and 17 years. There was no significant change in BMI category, EAT-26 score >20 or the prevalence of muscle gain practices across the four groups over time. The prevalence of low body esteem increased significantly in both boy (p = 0.002) and girl (p = 0.001) mixed ancestry adolescents. In addition there was a significant increase in the prevalence of weight loss practices in the black African girls (p = 0.018) and an increase in healthy weight control behaviors in the black African boys (p = 0.001). In contrast there was a 13.1% decrease in healthy weight control behaviors between the age of 13 and 17 years, in the mixed ancestry boys (p = 0.045).

**Table 3:** Longitudinal change between 13 and 17 year old urban South African _Adolescents._

Variables	Black Africanboys(n = 627)	P*	BlackAfricangirls(n = 675)	P*	MixedAncestralboys (n = 63)	P*	MixedAncestralgirls (n = 70)
**BMI cutoff**							
Underweight	**↑**4.1%	0.643	**↑**0.7%	0.084	**↑**1.6%	0.286	0%
Normal	**↑**1.6%		**↑**6.4%		**↓**3.2%		**↓**1.4%
Overweight	**↑**1.2%		**↓**4.5%		**↓**1.5%		**↓**1.4%
Obese	**↓**1.4%		**↓**2.5%		0%		0%
**EAT-26**							
>20	**↑**4.6%	0.529	**↓**4.9%	0.211	**↓**2.3%	0.822	**↓**0.6%
**Body esteem**							
Low	**↑**3.3%	0.09	**↑**3.4%	0.134	**↑**10.9%	**0.002**	**↑**12.3%
Average	0%		**↓**1.5%		**↓**9.7%		**↓**10.1%
High	**↓**3.3%		**↓**2.0%		**↓**1.2%		**↓**2.2%
**Weight control** **behaviour**							
Healthy	**↑**23.4%	**0.001**	**↓**1.2%	0.885	**↓**13.3%	**0.045**	**↑**7.3%

P*- P value for the longitudinal change in each ethnic and gender group.

### Female body silhouettes

The comparisons between the 17-year-old boys’ and girls’ perceptions of female body silhouettes are presented in Table 4. There was agreement between the boys and girls for most of the attributes with the majority of boys and girls associating the normal weight silhouettes with being the ‘best’, ‘getting respect’ and being the ‘happiest’, while the obese silhouette was associated with the ‘worst’ and the ‘unhappiest’, and the underweight silhouette with the “weakest”. There was a significant difference between the boys and the girls with regard to the silhouette that they considered to be “clumsy” and the “strongest”, although the majority of both groups associated these attributes with the obese silhouette. A bimodal pattern was observed for ‘the less respect’ attribute in both the boys and the girls as they associated the underweight and the obese silhouettes with ‘less respect’.

**Table 4:** Perceptions of female body silhouettes by 17-year-old urban South African boys and girls.

Silhouette	Boys (n = 658)	Girls (n = 717)	P-value
**Best**			0.06
Underweight	43(6.5%)	65(9.1%)	
Normal	436(66.3%)	496(69.2%)	
Overweight	166(25.2%)	144(20.1%)	
Obese	13(2%)	12(1.7%)	
**Worst**			0.228
Underweight	173(26.2%)	159(22.1%)	
Normal	10(1.5%)	9(1.3%)	
Overweight	10(1.5%)	7(1%)	
Obese	467(70.8%)	541(75.6%)	
**Clumsy**			**0.001**
Underweight	212(32.1%)	199(27.6%)	
Normal	28(4.2%)	22(3.1%)	
Overweight	63(9.5%)	31(4.3%)	
Obese	358(54.2%)	465(64.9%)	
**Respect**			0.798
Underweight	88(13.4%)	94(13.1%)	
Normal	286(43.4%)	327(45.7%)	
Overweight	166(25.2%)	178(24.9%)	
Obese	119(18.1%)	117(16.3%)	
**Less respect**			**0.002**
Underweight	283(42.9%)	323(45.2%)	
Normal	51(7.7%)	24(3.4%)	
Overweight	45(6.8%)	38(5.3%)	
Obese	281(42.6%)	330(46.2%)	
**Strongest**			**0.001**
Underweight	21(3.2%)	38(5.3%)	
Normal	89(13.5%)	163(22.7%)	
Overweight	162(24.6%)	198(27.6%)	
Obese	388(58.8%)	318(44.4%)	
**Weakest**			0.65
Underweight	593(89.9%)	629(87.7%)	
Normal	19(2.9%)	26(3.6%)	
Overweight	7(1.1%)	8(1.1%)	
Obese	41(6.2%)	54(7.5%)	
**Happiest**			0.45
Underweight	68(10.3%)	64(8.9%)	
Normal	323(49.1%)	382(53.4%)	
Overweight	172(26.1%)	175(24.4%)	
Obese	95(14.4%)	95(13.3%)	
**Unhappiest**			0.748
Underweight	194(29.6%)	196(27.4%)	
Normal	32(4.9%)	32(4.5%)	
Overweight	33(5%)	34(4.8%)	
Obese	397(60.5%)	454(63.4%)	

## Discussion

We have shown in the cross-sectional component of our study a high prevalence of overweight and obesity among Black African girls, reaching 27.5% in the 17 year old girls. In both age groups, the prevalence of weight loss attempts was higher in girls, and muscle gain attempts were higher in boys, when compared to the opposite sex. At age 17 years both mixed ancestry boys and girls had lower body esteem than their black African peers, and the longitudinal data confirmed an increase in the prevalence of low body esteem in the mixed ancestry boys and girls with age. In addition, the desire to look better was the most common reason reported for engaging in weight control behaviors for all adolescents. Longitudinally, the prevalence of healthy weight control behaviors increased in the black African adolescent boys, but decreased in the mixed ancestry boys, while in the black African girls there was an increase in the prevalence of weight loss practices.

In low-middle income countries (LMICs), there has been a reduction in the prevalence of underweight and an increasing concern around the increase in the prevalence of overweight and obesity in childhood and adolescence [20]–[22]. Compared to previous South African studies, our study had a lower prevalence of overweight/obesity, two studies from rural and urban areas found a prevalence of >5% [23], [24]. Our findings are consistent with results from previous studies in other developing countries undergoing rapid socio-economic, cultural and nutritional transitions, and experiencing both under nutrition and over nutrition [25].

Our study showed an increase in muscle gain practices among the boys and weight loss practices among girls. This may be linked to the socio-cultural transition occurring in South Africa with adolescent boys working towards being leaner and more muscular, and girls wanting to be leaner to meet the societal expectations of an ideal body. Results from studies conducted in high-income countries (HICs) observe a similar trend with men engaging in muscle gain practices to achieve a male ideal V-shaped figure and girls engaging in weight loss practices to attain an ideal lean body shape with an emphasis on slim hips, bottom, and thighs [26].

The prevalence of low body esteem increased between 13 and 17 years of age in both the mixed ancestral boys and girls, and at the age of 17 years the prevalence was higher in the mixed ancestral group compared to their black African peers. This could be as a result of the mixed ancestral adolescents not feeling like they belong to a particular ethnic group resulting in a lack of identity. Our findings suggest that adolescents become more aware of themselves and their bodies, resulting in more negative perceptions, thoughts and feelings about their own body with increasing age. Previous studies have found that when persons of different cultural backgrounds internalize the Western norms of thinness as the ideal, a greater degree of disordered eating is observed [27], [28]. Weight control behaviors were prevalent among the study sample. Although the use of healthy weight control practices was common at 13 and 17 years of age, a significant proportion of 13 and 17 year olds engaged in unhealthy weight control behaviors. The prevalence of unhealthy weight control behaviors in our study was higher than those found in HICs by Neumark-Sztainer and others (15% in males and 37% in girls) [2], and it is likely that South African adolescents are increasingly being subjected to societal pressure to meet the ideal body size or shape as the country continues to move through an epidemiological transition. An increase in weight loss practices occurred among black African girls between 13 and 17 years of age. This could be a result of conflict between traditional cultural beliefs and ‘Western’ expectations, with black African urban teenagers embracing Western norms to fit in with the demands of Western culture. This suggests that acculturation is slowly gaining hold among black African adolescent girls and eroding the more traditional/cultural concepts of an overweight female being beautiful, healthy and affluent.

Our study found that late adolescent boys and girls associated an obese silhouette with negative attributes such as the “worst, clumsy and unhappy”. This suggests that there is a degree of social stigma associated with female obesity among males and females in urban South Africa. This indicates a greater pressure for adolescent girls to be thinner in order to meet the social expectations associated with leanness including beauty and attractiveness. Previous international studies have shown that weight stigma invokes psychological stress that might lead to depression, low self-esteem and body dissatisfaction [29]–[31]. Similarly, boys and girls associated more positive attributes with normal weight silhouettes. This might be as a result of increased Westernization that facilitates a shift in the societal expectations of its adolescents. In line with Brink’s findings, in Western culture leanness is associated with being healthy, attractive and in control whereas, overweight and obesity is associated with poor health, laziness and lack of personal will [32].

Our study also found that an underweight silhouette was associated with weakness, which might reflect an association with HIV/AIDS and tuberculosis (conditions that are very common in South Africa). [33] Consistent with previous studies, [36–40], our study found no significant difference in eating attitudes across gender and ethnicity, suggesting that the Western culture is cross cutting in the South African population and that all ethnicities are becoming more exposed to each other’s cultures. This may be attributed to increased urbanization post-apartheid. Bilali and others found girls to be more at risk of internalizing messages sent by media, which idealizes an ultra-thin body size and shape. These results in adolescent girls having a negative body image and coming up with unrealistic goals that they want to attain so as to gain the “ideal” body shape and size. In the process of achieving these, they engage in unhealthy and extreme weight control behaviors [34].

The study has several strengths. We present longitudinal data and therefore we are able to illustrate the longitudinal change of psychosocial behaviors during adolescent maturation. Few studies in South Africa have included boys in their samples, thus our data on boys is unique and of value in helping to understand societal changes in adolescent attitudes. Further, the tools used in this study (EAT-26, body esteem and body silhouette) have been validated both internationally and within South Africa. This study also explored multiple methods i.e. EAT-26, body esteem and weight control behaviors, to assess adolescents’ behavior.

This study has several limitations; 1) the study only included black African and mixed ancestral participants due to the small numbers of responses from the other ethnic groups. It would be of value to investigate eating attitudes, body esteem and weight control behaviors across all the ethnic groups in South Africa. 2) The EAT-26 and body-esteem tool were self-reported and thus were dependent on participants’ honesty and accuracy. Social desirability bias may occur during the interviews due to interactions with the participants who may then over-report desirable behaviors and underreport undesirable behaviors. Recall bias may also occur in the interview setting due to the immediacy of the response required and the respondent alone has to judge whether the information they have recalled is relevant to the question and how best to respond.

## Conclusions

Black African girls had a higher BMI and an increased risk of developing eating disorders than girls of mixed ancestral origin. More adolescent girls engaged in weight loss practices than boys, whereas more boys engaged in muscle gain practices than girls. Weight control behaviors are prevalent among South African adolescents. In addition, low body esteem was prevalent in mixed ancestry adolescents. Adolescent boys and girls of both ethnic groups shared similar views on many of the female body silhouette attributes, which are similar to those expected in a western community.
