# Correction: Changes in Eating Attitudes, Body Esteem and Weight Control Behaviours during Adolescence in a South African Cohort

**DOI:** 10.1371/journal.pone.0117879

**Published:** 2015-03-05

**Authors:** The PLOS ONE Staff

The following information is missing from the Funding section: We would like to thank the Wellcome Trust for funding this project, reference no. 092097/Z/10/Z. The funder did not play a role in the study design, data collection and analysis, decision to publish, or preparation of the manuscript.

There are errors in Fig. 1. Please see the corrected version of Fig. 1 here.

**Fig 1:**
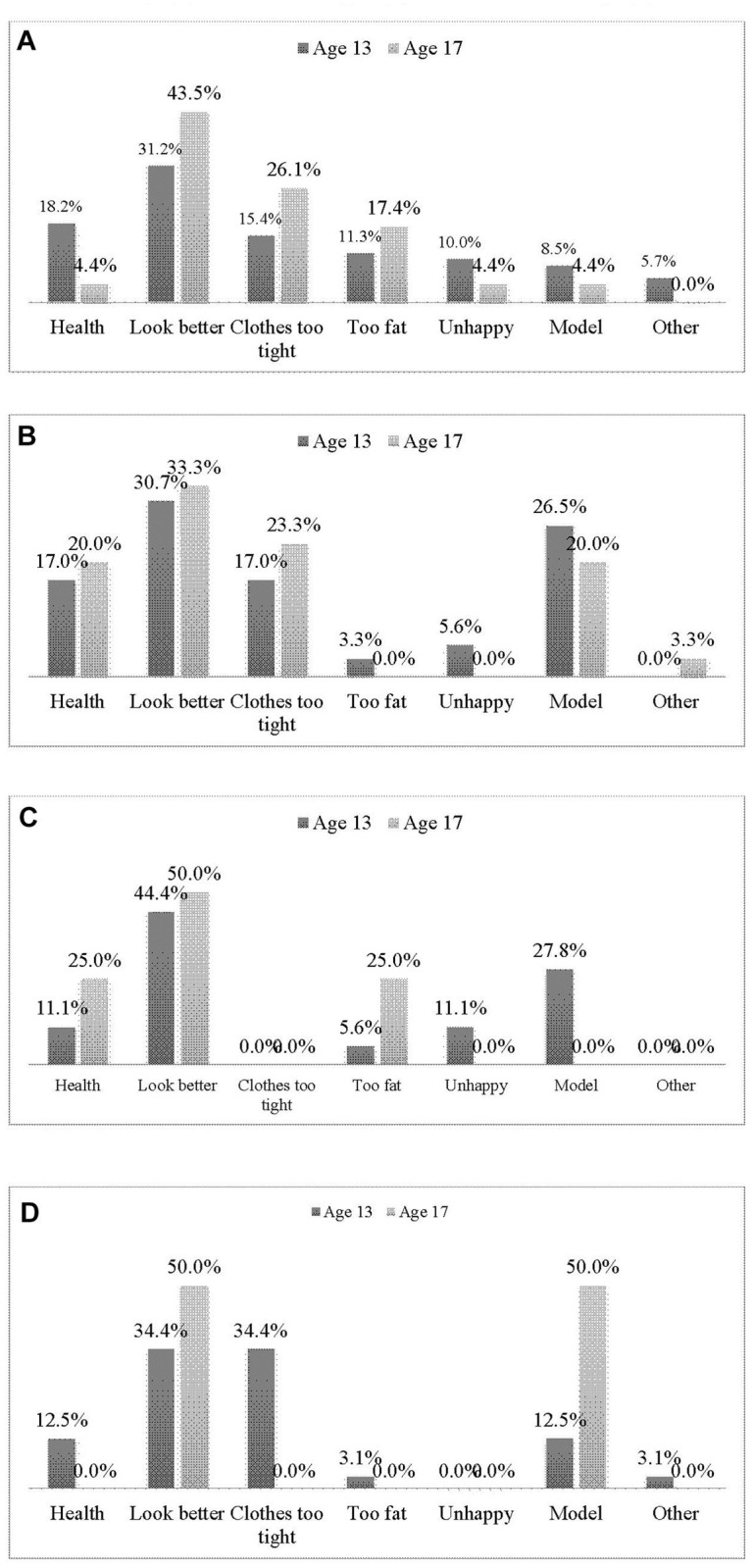
Reasons for weight control behaviors among age 13 and 17 black African girls (A); black African boys (B); mixed ancestral girls (C) and mixed ancestral boys (D).
